# Editorial: Physical exercise and other health behaviours in the management of breast cancer patients

**DOI:** 10.3389/fspor.2023.1357910

**Published:** 2024-01-08

**Authors:** Cassiano Merussi Neiva, Dalton Muller Pessoa Filho, María Marcela Gonzáles Gross

**Affiliations:** ^1^ Faculty of Sciences, São Paulo State University, Bauru, Brazil; ^2^ College of Medicine of University of Ribeirao Preto, Ribeirão Preto, Brazil; ^3^ INEF—Facultad de Ciencias de la Actividad Física y del Deporte: Departamento de Salud y Rendimiento Humano, Polytechnic University of Madrid, Madrid, Spain

**Keywords:** physical activity, breast cancer, active living, prevention and treatment, health behaviors

**Editorial on the Research Topic** Physical exercise and other health behaviors in the management of breast cancer patients

Breast cancer continues to be one of the main causes of morbidity and mortality among women worldwide. In this context, the approach to breast cancer management has evolved considerably in recent decades, integrating multidisciplinary strategies that aim not only to treat the disease, but also to promote the overall health of patients.

Adjuvant models in the classic treatment of different forms of cancer are increasingly discussed and used, especially in the treatment of patients with breast cancer, which is a form of the disease with peculiar effects because, in addition to all the physiological damage caused, it also brings discomfort, anguish and emotional suffering to more women, strongly impacting patients' self-esteem.

A crucial component of this progress is the growing recognition of the fundamental role of physical exercise and other health behaviours in the effective treatment of breast cancer. Scientific studies have consistently demonstrated the positive benefits of physical exercise in improving quality of life, reducing the risk of recurrence and mitigating the collateral effects associated with breast cancer treatment.

Our Research Topic “Physical Exercise and other Health Behaviours in the Management of Breast Cancer Patients”, sought to understand more about physical exercise and other health behaviours in the treatment of breast cancer and how this affects the quality of life of patients, hoping to lead to an effort to increase prevention, bring more effectiveness to treatment and cooperate with access to evidence-based medicine, thus improving the quality of life of breast cancer patients.

Regular physical exercise, adapted to the individual needs of patients, has shown significant impacts in reducing fatigue, improving physical function and reducing symptoms associated with therapy.

The articles published on the topic have contributed greatly to the advancement of knowledge in the field. The article entitled “Bibliometric analysis of global research on physical activity and sedentary behaviour in the context of cancer”, made an important contribution to the topic by analysing publications related to sedentary behaviour and physical activity in the context of cancer and summarising the most influential authors, countries, institutions and journals.

In summary, the results of the study show that exercise oncology is a broad research topic that focuses on cancer prevention, management and supportive treatment.

The study focussed on exercise and sedentary lifestyle in breast cancer patients and the role of Physical Activity in improving quality of life in survivorship. Emerging research focuses have generally been around prehabilitation programmes for cancer and remote intervention issues for physical activity.

In addition, the study helped to support the prioritisation of research topics in this field, as it provided information on gaps and deficiencies in the current literature.

In turn, the study “Meta-analysis of effects of yoga exercise intervention on sleep quality in breast cancer patients”, which sought to systematically evaluate and test the effects of yoga exercise intervention programmes on sleep quality in breast cancer patients in order to suggest more optimised exercise programmes, made a valuable contribution by pointing to evidence suggesting that yoga exercise intervention has good effects on improving sleep quality in breast cancer patients.

The type of positive meditation intervention, the frequency of the intervention of two or three times a week, the total duration of the intervention of 6 to 8 weeks and the evaluation immediately after the end of the intervention are pointed out as effective in improving sleep quality.

In the same vein, the article “Effects of Tai Chi Chuan training on the QoL and psychological well-being in female patients with breast cancer: a systematic review of randomised controlled trials”, which aimed to assess the effects of Tai Chi Chuan (TCC) on quality of life and psychological symptoms in women with breast cancer, showed that TCC-based exercises are useful for improving quality of life, anxiety and fatigue in breast cancer patients within the range of comparisons covered by this study.

Finally, the study “The effectiveness of exercise on the symptoms in breast cancer patients undergoing adjuvant treatment: an umbrella review of systematic reviews and meta-analyses”, through an umbrella review aimed at analysing current research evidence on the effectiveness of exercise in symptom control in breast cancer patients undergoing adjuvant treatment, pointed out that research is still needed to confirm the recommendations of most studies on exercise during adjuvant treatment of breast cancer patients, as it is fundamental for symptom management in the rehabilitation process.

The study also suggests that, in order to increase the accuracy of studies analysing the efficiency of exercise in symptom control, future studies may focus more on the application of bridge symptoms, symptom networks, and ecological instantaneous assessment.

In addition to physical exercise, other health behaviours also play a crucial role in the management of breast cancer. Eating a balanced diet, managing stress and promoting healthy habits such as smoking cessation have been associated with better treatment outcomes and quality of life for patients. It is imperative that health professionals, researchers and patients collaborate to integrate these positive health behaviours into the overall treatment plan.

Personalised strategies, adapted to the individual characteristics of each patient, should be developed to optimise adherence to these beneficial behaviours.

However, it is essential to recognise the challenges that can arise in implementing these strategies, including socioeconomic barriers, access to resources and the need for psychosocial support.

Therefore, continued research and the promotion of health policies that facilitate the incorporation of these behaviours into breast cancer management are essential.

In conclusion, the incorporation of physical exercise and other health behaviours into breast cancer management represents a significant advance in the holistic approach to this disease. Continued commitment to research, education and practical implementation of these strategies is key to improving clinical outcomes and quality of life for breast cancer patients. In doing so, we can't only treat the disease, but also empower women to live full and healthy lives after a breast cancer diagnosis.

